# Biological Potential of Silver Nanoparticles Mediated by *Leucophyllum frutescens* and *Russelia equisetiformis* Extracts

**DOI:** 10.3390/nano11082098

**Published:** 2021-08-18

**Authors:** Afrah E. Mohammed, Wafa Abdullah Al-Megrin

**Affiliations:** Department of Biology, College of Science, Princess Nourah Bint Abdulrahman University, Riyadh 84428, Saudi Arabia; Waalmegrin@pnu.edu.sa

**Keywords:** nanostructure, phytofabrication, apoptosis, antibacterial, antibiotic-nano composite

## Abstract

Awareness about environmental concerns is increasing, specially the pollution resulting from nanoparticles (NPs) production, which has led to great interest in the usage of biogenic agents for their fabrication. The current investigation used eco-friendly organic phytomolecules from *Leucophyllum frutescens* and *Russelia equisetiformis* leaves extract for the first time in the fabrication of silver NPs from silver ions and further an assessment of their biological activities was performed. The leaves extract from both plant sources were used as capping and reducing agents and added to AgNO_3_. The mixtures were observed for colour changes, and after a stable dark brown colour was obtained, the NPs were separated and further investigated using dynamic light scattering, transmission electron microscopy and energy-dispersive X-ray spectroscopy. The Fourier transform infrared spectroscopy technique was employed to determine the active organic ingredients in the plant extracts. The prepared NPs were tested against three cell lines (two cancer ones and one normal control) and the effects observed using TEM and confocal laser scanning microscopy (LSM). Antibacterial activity against two Gram positive and two Gram negative species was examined and the synergistic effect of the ampicillin-NPs conjugate was studied. Findings showed successful conversion of Ag ions into L-AgNPs and R-AgNPs achieved using *L.* *frutescens and R. equisetiformis* extracts, respectively. A mean size of 112.9 nm for L-AgNPs and 151.7 nm for R-AgNPs and negative zeta potentials were noted. TEM analysis showed spherical NPs and EDS indicated Ag at 3 keV. Reduction in cancer cell viability with low half-maximal inhibitory concentrations was noted for both tested NPs. Structural changes and apoptotic features in the treated cancer cell lines were noted by TEM and cell death was confirmed by LSM. Furthermore, higher antibacterial activity was noticed against Gram positive compared with Gram negative bacteria as well as high synergistic effect was noted for the Amp-NPs conjugate, specially against Gram positive bacteria. The current investigation has thus developed an eco-friendly NPs synthesis route by applying plant extracts to efficiently produce NPs endowed with potential cytotoxic and antibacterial capacity, which therefore could be recommended as new approaches to overcome human diseases with minimal environmental impact.

## 1. Introduction

Nanotechnology is the technology concerned with particles that range between 1–100 nm in size, their synthesis, description, as well as their usage [1]. Nanoparticles have a wide range of applications in the biomedical field. Nanomaterials display totally different properties from their bulk materials such as size, shape and distribution as well as high surface area that enable their attachment with several functional ligands [2]. Their small size facilitates their applications in biomedical fields [3,4], where some metal nanoparticles have been employed for diagnostic imaging, drug delivery, labelling, and as biosensors, as well as antimicrobial and cytotoxic agents due to their high compatibility with biological systems [5,6]. Generally, nanomaterials have special mechanical, magnetic, electronic and optical characteristics, thermal and catalytic potentials facilitating their medical applications [7,8]. One of the biggest medical concerns nowadays are infectious diseases caused by pathogenic bacteria. The multidrug-resistant (MDR) bacteria have the ability to adapted well to antibacterial agents. Using their different virulence factors, bacteria can suppress the efficiency of antibiotics and therefore plans to mitigate the development of MDR bacteria are urgently needed. In relation to well-known and used antibiotics, antimicrobial nanoparticles have various advantages such as the ability to overcome microbial resistance, potential for critical toxicity reduction as well as the fact such agents are cost efficient [9,10]. NPs can rapidly detect and treat microbial diseases [11] because their small size enhances penetration inside bacterial cells leading to cell destruction [12]. Therefore, nano-antibiotics could be a good alternative to currently used antimicrobial agents in alleviating the threat of MDR bacteria. Additionally, mortality due to cancer is increasing worldwide and therefore it poses a substantial challenge for efficient medications [13]. Since development of chemotherapeutic resistance in cancer cells is expected, seeking alternatives is an urgent issue. NPs could be efficient therapeutic agents in cancer treatment [14]. The Food and Drug Administration (FDA) permits the application of nanomaterials in human medicines in the USA [15]. On the other hand, regarding NPs synthesis, UV irradiation and radiolysis are some examples of physical synthesis methods for nanoparticle formation, besides evaporation and condensation. Nevertheless, these processes mostly require complex devices and high power therefore, high operating expenses can be expected [16]. Hydrogen, sodium borohydride and hydrazine are so of the reducing agents used in the fabrication of NPs [17]. In addition natural molecules like chitosan, rubber and cellulose are often used as reducing in the chemical synthesis of nanoparticles however, using such chemicals necessitate the addition of organic solvents due to their hydrophobic nature [16]. Using such chemicals may pollute the nanoparticles surface due to their toxicity, which might hinder their usage in some biomedical applications [18]. Such disadvantages of chemical and physical approaches suggest a need to look for alternatives for nanoparticles’ synthesis. Plant extracts and microorganisms have extensively used for biological syntheses of nanoparticles via green chemistry routes and are considered eco-friendly techniques with high compatibility for biomedical usage [19]. Biologically synthesized nanoparticles are defined by better morphology and size when compared with those resulting from other production methods [20] and in addition, the biomolecules from biogenic agents contribute to NPs’ stability via capping them and inhibiting their agglomeration [21]. Plants are widely used due to their availability and there are no limitations for their usage in microorganism applications. Previous studies used varied plant parts for the synthesis of silver nanoparticles and tested their ability against microbes and cancer cell lines [22,23,24,25]. The toxic effect of Ag ions in the human body is well documented but the toxicological characteristics of AgNPs might differ from those of their bulk materials [26], therefore, the synthesis of nanocomposites with less toxicity is needed. Biological approaches in NPs synthesis might be helpful in reducing the toxicity since they lead to functional particle surfaces due to high affinity of the natural molecules for NPs [27]. Capping of the NPs by the phytochemicals could be the main reason for the minimization of their toxic properties since stable, functional and safe AgNPs were produced when Ag ions were combined with biocompatible molecules of plant origin [28]. Varied medicinal plants were investigated in relation to nanoparticle formation however, different plant types and parts are expected to provide nanoparticles with varied characteristics. Such differences could be related to variations in the plant biomolecules that playing a significant role in capping and reducing metal ions to nanoparticles. Therefore, the current study aimed to use extracts of *Russelia equisetiformis* and *Leucophyllum frutescens* as capping and reducing agents in silver nanoparticle formation. *Leucophyllum frutescens* (Berl.) belongs to the family Scrophulariaceae [29] and is perennial bedding shrub that tolerates summer heat and drought stress conditions [30]. Recently powdery mildew caused by *P. xanthii* was reported for the first-time in *L. frutescens* during spring 2019 and 2020 [31]. These shrubs proved their ability to treating lung infections such as TB [32]. The perennial shrub, *Russelia equisetiformis* (firecracker) also belongs to the family Scrophulariaceae. It is an attractive plant with green stems that is easily cultivated from rooted cuttings [33,34] and available in varied areas. Mites, nematodes and chewing insects are the pests and diseases that most attack *Russelia equisetiformis* [35]. The plant was well documented as a source of anti-inflammatory and analgesic agents with membrane-stabilizing characteristics [36]. The plants under investigation are used as medicinal plants and their activities well proved and documented, however, no study was conducted in relation to their ability as biogenic agents in silver nanoparticle formation. Therefore, our study should be considered as the first report investigating *Russelia equisetiformis* and *Leucophyllum frutescens* extracts as biomediators in AgNPs formation. Furthermore, the prepared biogenic NPs were also investigated for their antimicrobial and cytotoxic potentials, as well as an assessment of the mechanism behind their cytotoxic abilities using TEM and LSM.

## 2. Materials and Methods

### 2.1. Plant Materials

*Russelia equisetiformis* and *Leucophyllum frutescens* leaves were collected from the nursery of the Royal Commission for Riyadh City (Riyadh, Saudi Arabia) during October 2020 from a 6-month age plants. Collected plants were identified and verified at Princess Nourah Bint Abdulrahman University (Riyadh, Saudi Arabia). Plant leaves were cleaned with distilled water, air dried, and then ground into a fine powder using a milling machine (IKA Werke GMBH and Co., Staufen im Breisgau, Germany). The powder was stored at room temperature in plastic bags until use.

### 2.2. Preparation of Aqueous Extracts for AgNPs Synthesis

Aqueous extracts from the leaves were prepared by the addition of distilled water to the plant powder at a ratio of 100:2 (*v*:*w*). The mixture was heated for 15 min (90 °C), and Whatman No. 1 filter paper (diameter of 125 mm) was used for filtration. Afterwards the filtrate (10 mL) was added to 1 mM AgNO_3_ (90 mL) and again heated for 10 min at 90 °C. The reaction medium was covered with aluminium foil and kept in the dark at room temperature and the colour change was observed until a stable dark brown colour was obtained, indicative of the formation of Ag NPs. Afterward, the mixture was centrifuged for 20 min at 14,000 rpm, the supernatant was discarded and the pellet was washed two times with distilled water repeating the same conditions and then kept for drying at room temperature. Finally, 1 mg/mL of each NPs were kept for further investigations.

### 2.3. NPs Characterization 

Different methods were used for the description of the prepared biogenic NPs. Hydrodynamic size and zeta potential analysis were determined by a dynamic light scattering (DLS) system using a Zetasizer (NANO ZSP, Serial Number: MAL1034318, ver 7.11, Malvern Instruments Ltd., Malvern, UK). For the size distribution and morphology analysis, electron transmission microscopy (TEM) was applied using a TEM system (JEM-1011, JEOL, Tokyo, Japan) at 80 kV voltage. A drop of NPs solution was taken and laid on a carbon-coated copper grid (200 mesh) and further placed in a vacuum desiccator for drying [37]. Energy-dispersive X-ray spectroscopy (EDS) was applied using a scanning electron microscope (JEOL JED-2200 series) to examine the NPs’ surface and further prove the presence of elemental silver [38]. 

### 2.4. Analysis of Surface Functional Groups 

To validate the probable organic molecules in the plant extracts as well as the biogenic NPs’ solutions, Fourier-transform infrared spectroscopy (FTIR) examination was done using a FTIR spectrometer (SPECTRUM100, PerkinElmer, Wellesley, MA, USA), at 450–3500 cm^−1^ employing a diffuse reflectance attachment [39]. 

### 2.5. Antibacterial Screening 

Antibacterial susceptibility evaluation was performed using the agar well diffusion procedure. two Gram-positive bacteria strains: methicillin-resistant *Staphylococcus aureus* (MRSA) and *Streptococcus mutans*, and two Gram-negative ones: *Klebsiella pneumoniae* and *Escherichia coli* were taken to test the antibacterial effect of the biogenic NPs. Microorganisms were obtained from the Bio-house medical lab (Riyadh, Saudi Arabia). For each strain, suspensions in saline were prepared at 0.5 McFarland concentration (1.5 × 10^8^ CFU/mL) by the direct colony suspension approach. Then bacteria were inoculated on agar plates and wells were prepared for NPs addition and 40 µL of 1 mg/mL NPs was added to each well separately and kept for I h under aseptic conditions for drying. Distilled water was used as a negative control, then plates were kept in a 37 °C incubator for 24 h. Afterwards, the clear zone around each well was measured in mm.

### 2.6. Minimum Inhibitory Concentration and Minimum Bactericidal Concentration 

The inhibitory and bactericidal properties of biogenic NPs were evaluated by the serial dilution technique. A volume of bacterial suspensions (100 μL/well) at 1.5 × 10^8^ CFU/mL were cultured in 96-well plates with various levels of each NPs concentrations (0.2, 0.5, 0.8, 1.1, 1.4, 1.7, 2.0, and 2.3 mg/mL) then kept at 37 °C incubator for 24 h. MIC and MBC values were determined by an agar well diffusion method.

### 2.7. Ampicillin-Conjugated NPs (Amp-NPs) Synthesis and Application

Ampicillin-nanocomposites (Amp-NPs) were prepared using a one-pot reaction as reported by Fan et al. [40] with minor modifications. One mL of ampicillin solution at a concentration of 1 mg/mL was mixed with 1 mL of each NPs solution at the same concentration. Then the mixture was kept for 48 h in the dark while shaking at room temperature, subsequently washed, and a solution of 1 mg/mL of Amp-nanocomposites was prepared for each NPs. Thereafter, the antibacterial activities were evaluated for the nanocomposites using the well diffusion method and the area around each well (mm) was measured after an incubation process for 24 h at 37 °C. 

### 2.8. Anticancer Action Induced by Biogenic NPs

The viability of the breast cancer cell line MDA-MB-231 and the colorectal cancer cell line HCT116 besides MCF 10A cells as a standard cell line was determined using an MTT assay. Cells were cultured at a concentration of 5 × 10^4^ cells/well at 96-well plate at 37 °C and at 95%/5% (humidified air/CO_2_). After 24 h, the media was removed and phenol-red free DMEM including 0.5% fetal bovine serum (FBS) was added. Thereafter, varied concentrations of prepared NPs were added to the cells, which were then kept for 48 h, the media was then removed and the cells were washed with PBS. MTT tests were accomplished at 570 nm absorbance using a Spectra Max microplate absorbance reader (Molecular Devices, San Jose, CA, USA) [41]. Additionally, for apoptosis assessment, LSM and TEM analyses were used to examine treated MDA-MB-231 cells.

### 2.9. Ultrastructural Cell Changes by Transmission Electron Microscopy

Untreated and NPs-treated MDA-MB-231 cells were prepared for investigation under TEM. First cell sections were prepared according to Ali et al. [41] with slight modifications, then these were loaded on a grid (Product G200-Cu, EMS, Ottawa, Ontario, Canada) and 1% uranyl acetate (Product 93-2840, STREM CHEMICALS, Newburyport, MA, USA) was applied in the dark for staining (15 min.) then normal saline was used for washing six times and afterwards 0.5% lead citrate (Product 17810, EMS) was applied. Besides, several sodium hydroxide pellets were washed with distilled water. Subsequent to drying, samples were evaluated using a TEM system (JEOL JEM 1400).

### 2.10. Laser Scanning Microscopy (LSM)

MDA-MB-231 cells were loaded in an eight well dish (Ibidi, Munich, Germany) 24 h before treatment. Then they were subjected to L-AgNPs (18 μg/mL) and R-AgNPs (7 μg/mL) treatment and then incubated under CO_2_ (5%) for 24 h at 37 °C. Subsequently, Calcein AM (Green), HOECHST 33342 (Blue) nucleus and PI (Red) staining were applied for live cells, nuclei and dead cells, respectively. An LSM780 microscope system (Zeiss, Jena, Germany) was used for imaging using a UV laser diode at 350 nm/460 nm for HOECST 33342, an argon laser at 488 nm/530 nm for Calcein AM, and an Intune laser at 90 nm/640 nm for PI.

### 2.11. Statistical Analysis

All assessments were completed for three replicates and results are displayed as mean ± standard deviation (SD). Variations among the studied parameters were statistically obtained by one-way analysis of variance (ANOVA) using the Prism 9.1 software (GraphPad Software Inc., La Jolla, CA, USA) for the half-maximal inhibitory concentration (IC_50_) graphs. Significance of the data is shown at *p* < 0.0001, *p* = 0.001, and *p* < 0.01.

## 3. Results and Discussion

**Figure 1:**
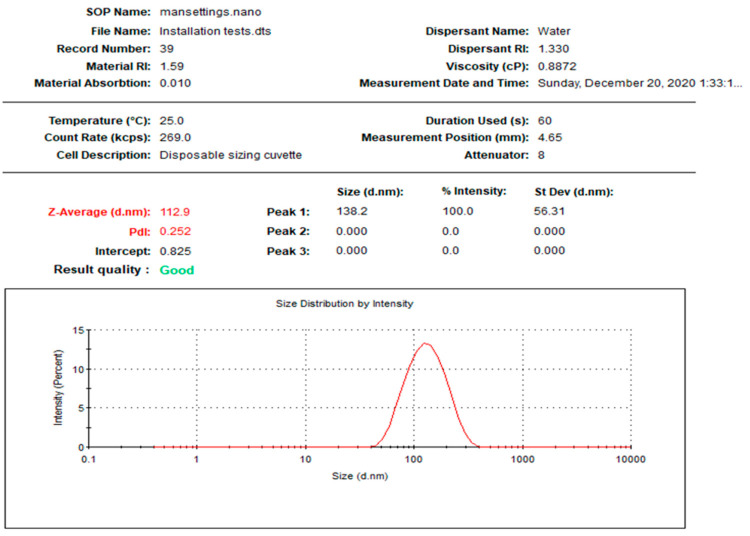
Size distribution of L-AgNPs.

**Figure 2:**
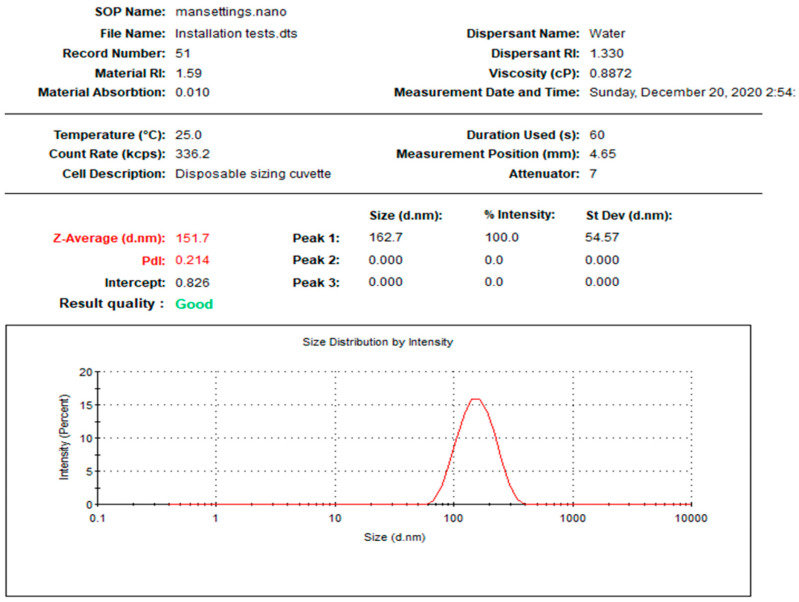
Size distribution of R-AgNPs.

In the current study, NPs were prepared using two different biogenic agents, *Leucophyllum frutescens* and *Russelia equisetiformis* leaves extracts, providing L-AgNPs and R-AgNPs, respectively. The first observation that proved the successful fabrication of NPs was the colour of the AgNO_3_, that changed to dark brown after 8 h and 24 h when combined with *L. frutescens* and *R. equisetiformis* extracts, respectively. The colour intensity was observed to increase in a time-dependent manner until no further changes were noted which was considered as a sign of the total biotransformation of Ag^+^ ions into Ag^0^. Thisbrown colour is characteristic of the plasmon vibration excitation on the surface of AgNPs [42]. Following the separation of the prepared NPs, 1 mg/mL was taken for further investigations. Figure 1 and Figure 2 indicate the size distribution the NPs, showing mean sizes of 112.9 and 151.7 nm for L-AgNPs and R-AgNPs, respectively, with polydispersity indices (PDI) of 0.252 and 0.214 (Table 1). Size was slightly varied between both prepared NPs suggesting different biomolecules from each extract since phytochemicals in the extract are the responsible agents in NPs reduction and stabilizing [43].

**Figure 3:**
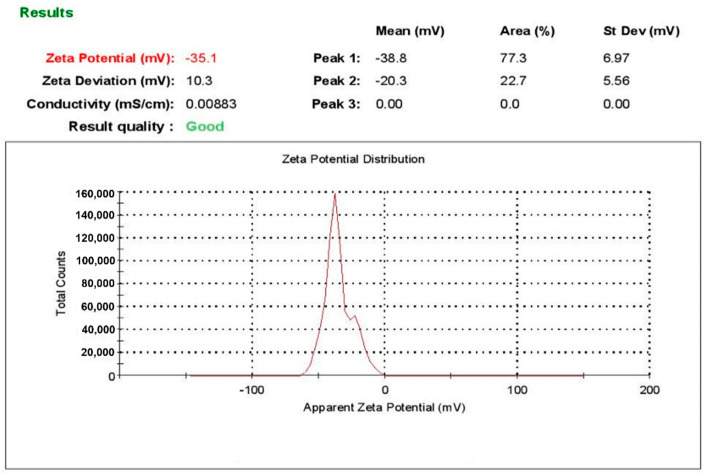
Zeta potential of L-AgNPs.

**Figure 4:**
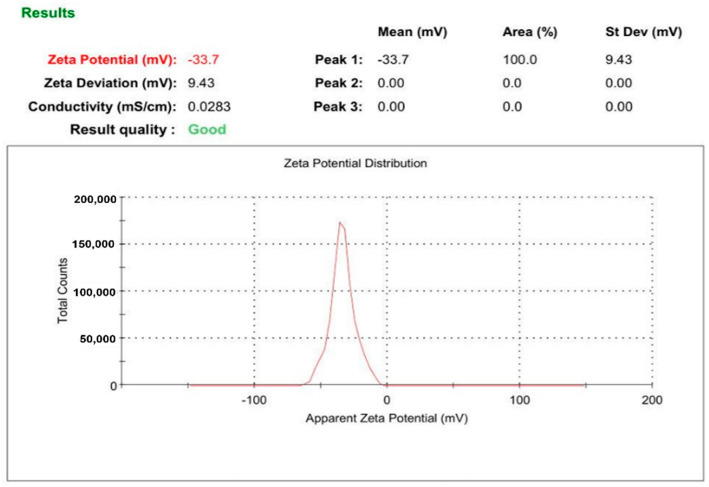
Zeta potential of R-AgNPs.

Furthermore, zeta potential measurements produced values of −35.1 and −33.7 mV for L-AgNPs and R-AgNPs, respectively (Figure 3 and Figure 4). Various reports have indicated negative zeta potentials for biogenic NPs fabricated using plant materials [23,44]. Negative low potential values might be related to phytochemicals with negatively charged functional groups that cap the NPs leading to electrostatic repulsion between the particles and therefore, NPs with good stability can be expected [45]. 

**Figure 5:**
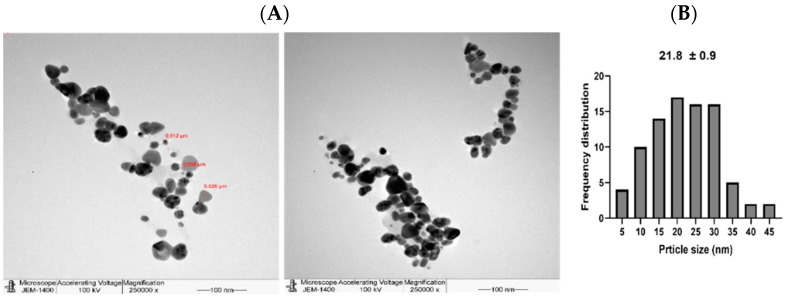
Spherical shape L-AgNPs (**A**) and their frequency distribution with mean particle size (**B**). Size measurements were analyzed by ImageJ software constructed from TEM micrographs at scale bars: 100 nm.

**Figure 6:**
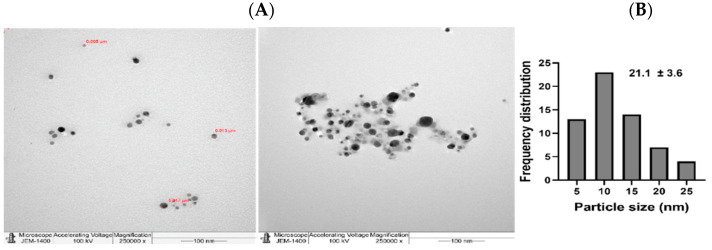
Spherical shape R-AgNPs (**A**) and their frequency distribution with mean particle size (**B**). Size measurements were analyzed by ImageJ software constructed from TEM micrographs at scale bars: 100 nm.

**Figure 7:**
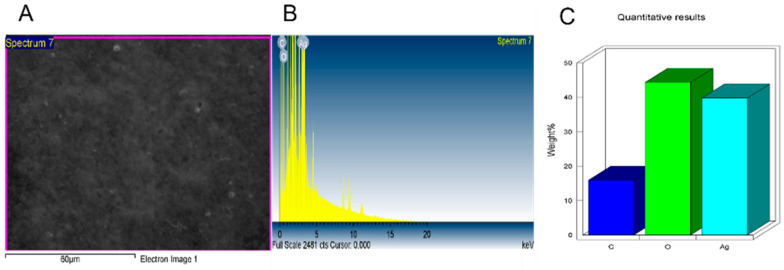
Surface morphology of L-AgNPs (**A**) and quantitative data analysis of images, weights of the of carbon, oxygen and silver atoms using EDS (**B**,**C**).

**Figure 8:**
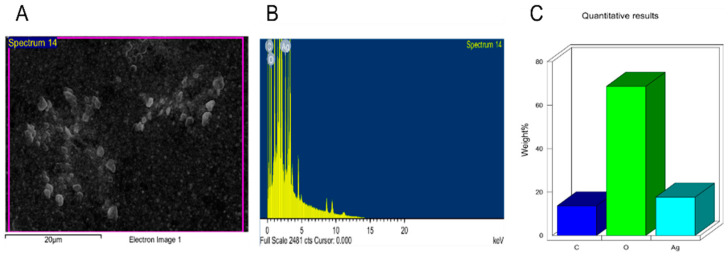
Surface morphology of R-AgNPs (**A**) and quantitative data analysis of images and describing the weights of the of carbon, oxygen and silver atoms using EDS (**B**,**C**).

Additionally, the NPs were further tested under TEM (Figure 5 and Figure 6) where good distribution and spherical shapes were seen, with mean size diameters of 21.8 and 21.1 nm for L-AgNPs and R-AgNPs, respectively, calculated using the ImageJ software. The higher hydrodynamic size of NPs than that detected by TEM could be the result of some impurities in the plant biomolecules that cap the NPs. However, both techniques showed nanosized L-AgNPs and R-AgNPs. Quantitative analysis of elements was perfomed by EDX analysis and the presences of Ag, C and O were verified, where the Ag signals at 3 keV and well distributed NPs were observed (Figure 7 and Figure 8). No particle-to-particle adherence and agglomeration was proved by either TEM of SEM analysis which might be a resulted of repulsion among the NPs due to the plant active ingredients that cap the NPs. The utilization of plant extract molecules in the NPs’ formation could also be indicated by the presence of C and O in NPs film noted by the EDS technique.

**Figure 9:**
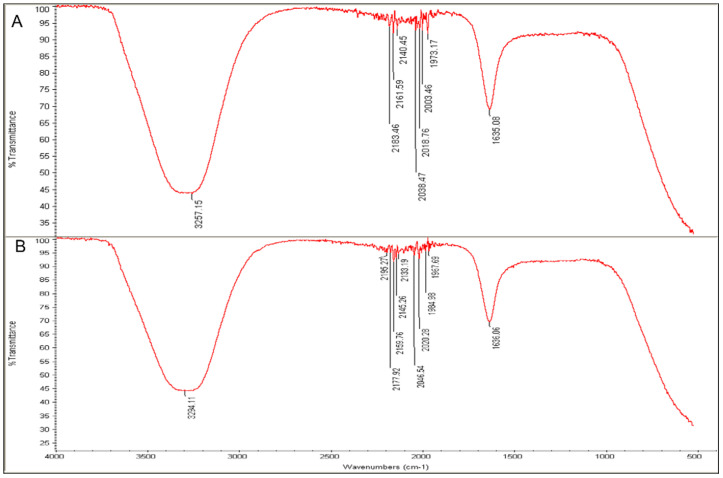
FTIR absorbance peaks of aqueous extracts of *L.* *frutescens* (**A**) and L-AgNPs (**B**) presenting peaks of the extract organic materials.

**Figure 10:**
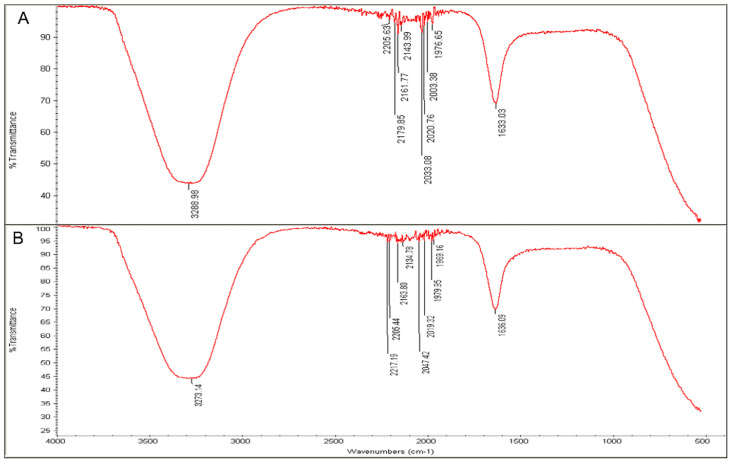
FTIR absorbance peaks of aqueous extracts of *R. equisetiformis* (**A**) and R-AgNPs (**B**) presenting peaks of the extract organic materials.

FTIR analysis was key for detecting the biomolecules that contributed to the fabrication of NPs from Ag ions since it is an analytical method that can identify organic and inorganic materials [46]. Figure 9 and Figure 10 present the major peaks noted at 3257.15 and 3288.98 cm^−1^ in the spectra of the L-extract and R-extract, respectively, indicating the presence of polyphenolic-OH groups and N-H stretching of amines as the detected values were approximately 3300 cm^−1^ [47]. Slight alterations in the peaks were noted to be 3294.11 and 3273.14 cm^−1^ for fabricated L-AgNPs and R-AgNPs, respectively. Other intense peaks were noted at 1635.08 and 1633.03 cm^−1^ for L-extract and R-extract, respectively, indicating amide 1 and carbonyl (C = O) stretching of proteins [46,48]. It was also observed that such values were slightly changed in the fabricated L-AgNPs and R-AgNPs solutions to 1636.06 and 1636.09 cm^−1^, respectively. The presence of varied biomolecules such as proteins and polyphenolics was noticed by several peaks in all tested solutions, demonstrating their role as capping and stabilizing agent in the AgNPs fabrication process. 

Although not investigated herein, the chemical components of both plants were identified previously. The natural serrulatane diterpenoids are well distributed in plants from the Scrophulariaceae family [49]. About 27 and 20 chemical compounds were identified in essential oils from *L.* *frutescens* and *R. equisetiformis*, respectively, by the GC-MS technique [33]. The current results displayed the ability of *L. frutescens* and *R. equisetiformis* extracts to provide nanosized particles in AgNPs fabrication, which might be due to their richness in chemical components. Therefore, more investigations were undertaken to address some biomedical applications such as anticancer and antimicrobial abilities since a large surface area is one of the characteristic features of NPs known to enhance their activity is these applications [50].

**Figure 11:**
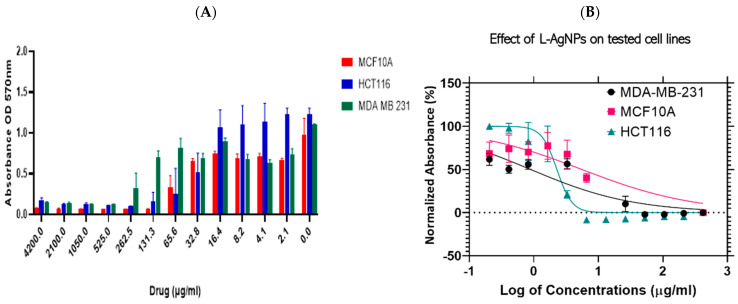
Effect of L- AgNPs on the cell viability of normal and cancer cells. Bar graph showing dose relationship of L-AgNPs on the viability of two human cancer cell lines; HCT116 (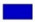) and MDA-MB-231 (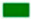) and a normal cell line; MCF 10A (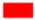) (**A**). Log dose-response relationship of L-AgNPs on the normalised viability of two human cancer cell lines; HCT116 (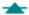) and MDA-MB-231 (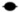) and one normal cell line MCF 10A (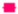) (**B**). IC_50_ values of AgNPs on each cell line was calculated using Log viability vs. normalised response–variable slope (four-parameter).

**Figure 12:**
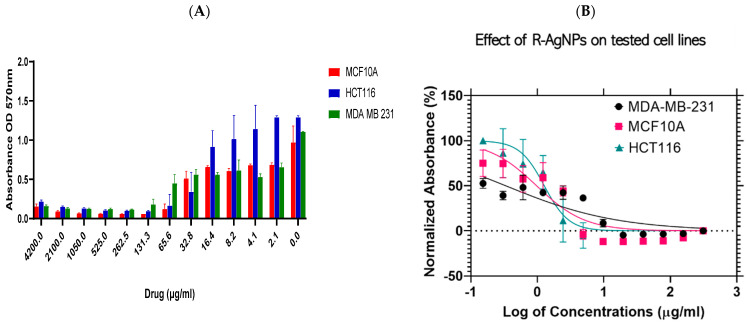
Effect of R- AgNPs on the cell viability of normal and cancer cells. Bar graph showing dose relationship of R-AgNPs on the viability of two human cancer cell lines; HCT116 (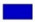) and MDA-MB-231 (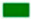) and a normal cell line; MCF 10A (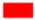) (**A**). Log dose-response relationship of R-AgNPs on the normalised viability of two human cancer cell lines; HCT116 (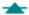) and MDA-MB-231 (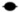) and one normal cell line MCF 10A (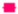) (**B**). IC_50_ values of AgNPs on each cell line was calculated using Log viability vs. normalised response–variable slope (four-parameter).

The efficiency of NPs was proved when tested against a triple-negative ER, PR-, HER2- human epithelial breast cancer lines (MDA-MB-231) and colorectal cancer cell lines (HCT116) in addition to a non-malignant human breast epithelial cell line (MCF 10A) that was used as a control by applying MTT assays. The results indicated a cytotoxic ability in a dose-dependent reaction manner since high NPs concentrations increased the cytotoxic effect as shown in Figure 11A and Figure 12A. Dose-dependant activity was noted for both tested NPs against the three cell lines tested. Furthermore, IC_50_ values were calculated using Log viability vs. normalised response–variable slope (four-parameter) and shown in Figure 11B for L-AgNPs and Figure 12B for R-AgNPs.

The biogenic NPs showed cytotoxic effects against all cell lines tested (Table 1) indicating a lowest IC_50_ of 8.8 and 3.3 μg/mL against MDA-MB-231 for L-AgNPs and R-AgNPs respectively. Moreover, the IC_50_ values for the colon cancer cell line were 22.8 and 12.7 μg/mL for L-AgNPs and R-AgNPs, respectively. Interestingly the highest IC_50_ against normal cells was noted for L-AgNPs (55.5 μg/mL) while R-AgNPs showed a low IC_50_ (9.7 μg/mL). Hereby, MDA-MB-231 and HCT116 could be selectively targeted since a very low concentration of L-AgNPs is needed to kill 50% of cells and a high concentration was needed to kill 50% of normal cells.

Furthermore, the current results indicated that R-AgNPs could selectively target only the MDA-MB-231 cell line since their toxic effect is greater on the normal breast epithelial cells than the colorectal cancer cell line. The toxicity of NPs against normal cell lines is a main reason behind their restriction for medical application for cancer treatment. NPs toxicity is highly associated with their ability to produce free radicals that attack cell biomolecules leading to cell function losses, necrosis, apoptosis and cell death [51]. R-AgNPs had better efficiency against both cancer cell lines in relation to the effect of L-AgNPs which might be related to variation in the plant metabolites that cap the NPs since their variation in the size is not highly significant. An earlier study concluded a hepatoprotective ability of *L. frutescens* ethanolic extract in liver damage induced by CCl_4_ in Wistar albino rats [52]. 2′,5″-Dimethoxysesamin isolated from the root bark of *L. frutescens* proved cytotoxic effects against Vero cells [32]. Furthermore, *R. equisetiformis* ethanolic extract was recommended to be used as folk medicine as an antimalarial drug [53] however, their methanol and aqueous extracts decreased normal rats’ liver function [54]. Moreover, *R. equisetiformis* extract showed a slight cytotoxic effect when their haemolytic action was studied against human red blood cells where a lysis ability was noted below 5.0% [33]. Interesting findings related to *R. equisetiformis* extract as pharmaceutical agent could be due to their active ingredients such as terpenoids, saponins, steroids, flavonoids, tannins, and alkaloids [33]. However, no report is available regarding its usage as reducing and capping agent in NP formation, and the same was also noted regarding *L. frutescens* extract.

**Figure 13:**
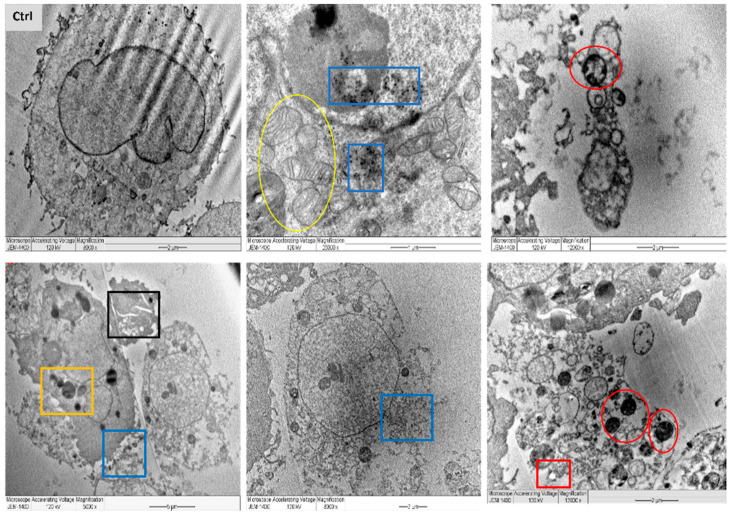
TEM images of MDA-MB-231 cell lines, where untreated cell (Ctrl) exhibiting rounded shape cell with complete organelle and nucleus. Image magnification ×8000; bar 2 μm. Other images at varied magnification show the ultrastructural alteration in cell treated with L-AgNPs displaying early apoptosis characteristics such as: chromatin condensation and over whole cell shrinkage as well as late apoptosis: lipid droplet (red square), peroxisomes (yellow square) and enlarged mitochondria (yellow circle) and damaged mitochondria (red circle). Damaged cancerous cells are clearly observed with R-AgNPs located in the cytoplasm, outer cell and nucleus membranes (blue squares).

**Figure 14:**
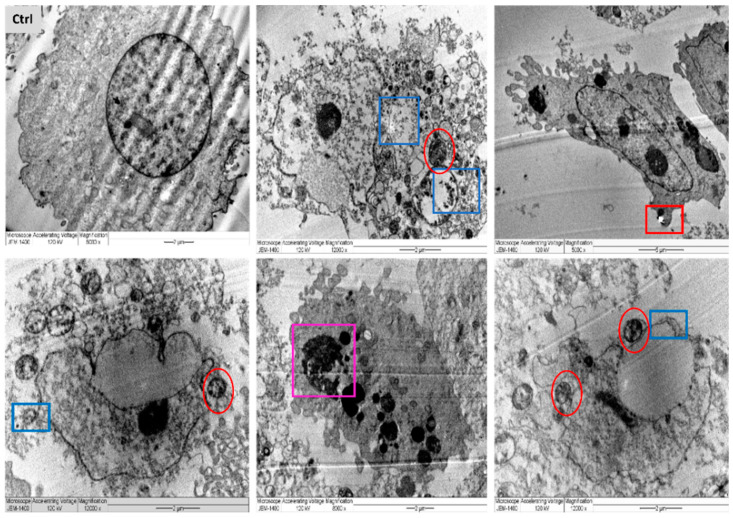
TEM images of MDA-MB-231 cell lines, where untreated cell (Ctrl) exhibiting rounded shape cell with complete organelle and nucleus. Image magnification ×6.000; bar 2 μm. Other images at varied magnification show the ultrastructural alteration in cell treated with R-AgNPs displaying early apoptosis characteristics such as: chromatin condensation and over whole cell shrinkage as well as late apoptosis: lipid droplet (red square), peroxisomes (yellow square) and enlarged mitochondria (yellow circle), damaged mitochondria (red circle) and condensed nucleus (Purple square). Damaged cancerous cells are clearly observed with R-AgNPs located in the cytoplasm, outer cell and nucleus membranes (blue squares).

In a trial to find out the exact mechanism of the L-AgNPs and R-AgNPs action on cancer cells, MDA-MB-231 cells were treated with the IC_50_ from NPs and tested under LSM and TEM. Variations between necrotic and apoptotic cell death can be assessed by electron microscopy as a useful approach for the analysis of the morphology of tumour cells [55]. Typical characteristics of cancer cells were noted, with compact cell membranes, entire cell organelles, clear nuclear membranes and well disseminated chromatin. Besides, microvilli and some vacuoles were noted for untreated MDA-MB-231 cells. However, the morphology of the NPs-treated cell lines was totally different in relation to control. TEM analysis presents NPs inside the cell cytoplasm and nuclei with irregular shapes and cell component leakage due to membrane damage besides less or absent microvilli. Lipid droplets, peroxisomes damage and enlarged mitochondria were also noticed, as well as enlarge nuclei and vacuoles beside cell shrinkage, chromatin condensation and mitochondria damage were also evident (Figure 13 and Figure 14). 

Ultrastructural alterations typical of early and late apoptosis in the treated cell lines were noticed for both treatments, suggesting same mechanism of action for both tested NPs. Observed cell damage could be related to the small size of the particles and the successful entry of NPs that deposited later in cell organelles and cytoplasm due to cell-NPs interactions leading to cell death. The interactions of the NPs with cancer cells resulted in their metastatic properties leading to reduced cell microvilli and destruction of the cell membranes [56]. A further explanation of the cell damage is the enhancement of oxidative stress by the NPs. Furthermore, the number of peroxisomes was increased as a reaction to overcome the oxidative stress since they have an ability to reduce the reactive oxygen species’ metabolism [57]. Easy penetration and accumulation of NPs inside cancer cell may enhance their application as anticancer therapy. Uptake of HeLa cells to Au@mSiO_2_ as a drug nanocarrier via cell endocytosis was also proved by Bio-TEM [58]. MCF-7 breast cancers showed similar damage features regarding early and late apoptosis when treated with CaCO_3_/Dox nanocrystals [59].

**Figure 15:**
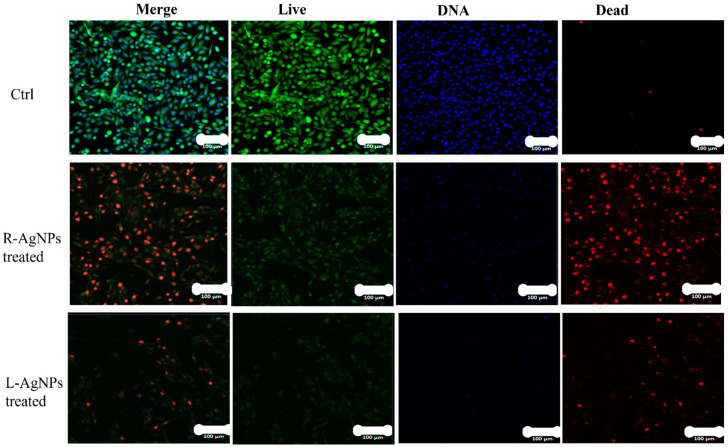
Confocal LSM imaging of L-AgNPs and R-AgNPs treated MDA-MB-231 cell lines as well as untreated control. Cells stained with Propidium Iodide, red (dead), Calcein AM, green (live) and HOECHST33342, blue (DNA). Overlay of all three stains (merge). Cells that had a bright green fluorescence were live cells. Cells with a bright blue colour indicated fragmented nuclei involving concentrated chromatin and were identified as apoptotic cells.

This possible mechanism was also confirmed by LSM (Figure 15). Following NPs treatments, MDA-MB-231 cells were investigated where apoptotic features of treated cell lines such as reduction in cell viability were noted, and necrotic signs were clear, since red colour PI stained DNA was abundant. Similar findings were noted on colon cancer cells when hyaluronic acid layered lipid-based chimeric NPs were applied [60]. Apoptosis of breast cancer cells was well documented for NPs promoting ROS, however, they can also enhance the proliferation of cancer and normal cells [61,62].

**Figure 16:**
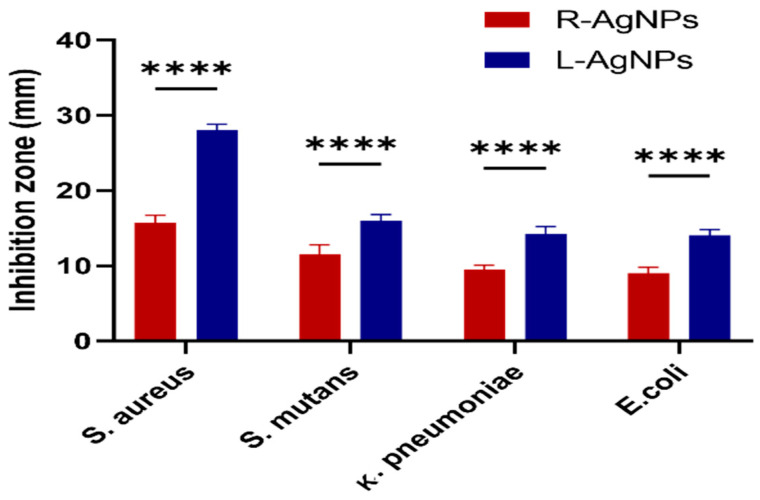
Antibacterial activity of L-AgNPs and R-AgNPs as inhibition zones (mm) against four different bacteria. Two-way ANOVA with multiple comparison was performed to identify differences between groups. *p* < 0.0001 (****).

Furthermore, the antibacterial ability of plant extracts and biogenic NPs was studied against *S. aureus*, *S. mutans, E. coli* and *K. pneumoniae* by the agar well diffusion method. The study outcomes are presented as the diameters in mm of the observed inhibition zones (Figure 16). Both plant extracts showed no activity against any of the tested bacteria however, high antibacterial activity of both NPs was noted against S. *aureus*, with an average inhibition zone diameter of 28 ± 0.8 mm and 15.8 ± 0.9 for L-AgNPs and R-AgNPs, respectively (*p* < 0.0001) followed by *S. mutans;* 16 ± 0.8 mm and 11.5 ± 1.2 for L-AgNPs and R-AgNPs, respectively (*p* < 0.0001). Lower activity was noted for Gram-negative microbes compared to Gram-positive ones, where 14.3 ± 0.9 and 9.5 ± 0.5 mm was noted against *K. pneumonia* (*p* < 0.0001) and 14.0 ± 0.8 mm and 9 ± 0.8 mm was noted against *E. coli* for L-AgNPs and R-AgNPs, respectively (*p* < 0.0001). It was noted that the activity of L-AgNPs was higher than that for R-AgNPs, indicating the same trend against the tested microbes. NPs’ activity against microbes is mainly related to their large surface area and small size since easy penetration via bacterial cell walls can be expected [50]. Furthermore, Menchaca et al. [63] indicated that a methanolic extract of *L. frutescens* had significant efficiency against *S. aureus* (MIC, 28.0 μg/mL) and *E. coli* (MIC, 30.0 μg/mL). A *n*-hexane extract of *L. frutescens* root showed antimycobacterial activity (MIC 63 μg/mL) against *Mycobacterium tuberculosis* [29]. *R. equisetiformis* has diverse medical applications due to its varied biological activity such as anti-inflammatory [64] antimicrobial, and antioxidant action, that might be related to its biomolecules [33]. Although the above findings proved the biological activities of *L. frutescens* and *R. equisetiformis* extracts our current findings indicated no antibacterial efficiency for the plant extract, which might be related to the low material concentration used or a high resistance ability of the tested microbes. Furthermore, lower MIC and MBC values were noted for Gram-positive bacteria (0.5 and 0.8 mg/mL, respectively) compared to those of Gram-negative bacteria (0.8 and 1.1 mg/mL, respectively) for both tested NPs, as displayed in Table 2. AgNPs are well known nano-antibiotics with high capability against infections resulting from resistant bacterial biofilms [65]. Recently Alqahtani et al. [66] proved the antibacterial ability of biogenic AgNPs mediated by lichen extract. Generally, NPs have an aptitude to induce ROS that damage cell macromolecules resulting in microbial death [67].

**Figure 17:**
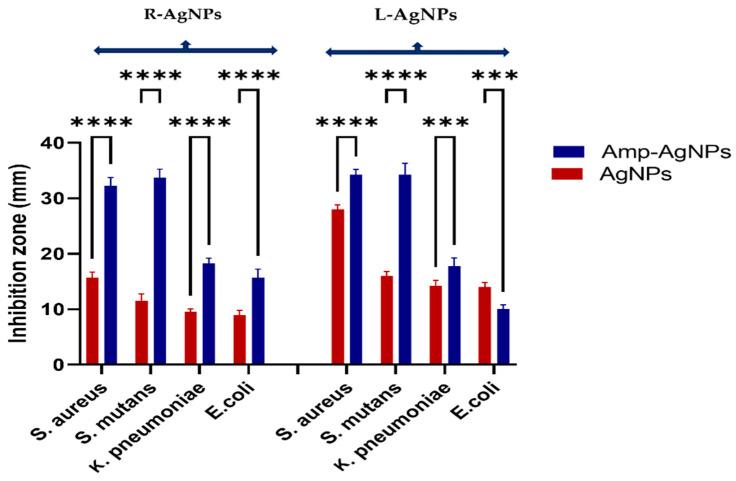
Antibacterial activity of AgNPs and Amp-AgNPs. Agar disc zonal inhibition method measuring antibacterial activity as inhibition zones (mm) of AgNPs and Amp-AgNPs against four different bacteria. Two-way ANOVA with multiple comparison was performed to identify differences between groups. *p* < 0.0001 (****), *p* < 0.001 (***).

On the other hand, for antimicrobial treatments, antibiotic-metal NPs conjugates could be efficient in treating resistant pathogens. The most commonly used metals as drug carriers are Zn, Au, Ag and Fe [68,69]. Consequently, in the current study Amp-NPs conjugates were tested as antibacterial agents and their activities were compared with the NPs and ampicillin. Significantly higher activity was noted for the Amp-R-AgNPs nanostructure compared with R-AgNPs activity (*p* < 0.0001) as presented in Figure 17. The same was also observed for L-AgNPs except for *E. coli* that showed higher tolerance to the conjugate in relation to NPs alone. It could be concluded that the combination with ampicillin resulted in a varied range of NPs antibacterial synergism such as in *S. mutans* that reached 100% synergism in Amp-R-AgNPs. The highest level of synergism of Am-NPs conjugate was noted against Gram-positive bacteria. The ability of AgNPs to be coupled and covered with antibiotics via their active groups was reported [70,71], therefore, a synergistic effect was expected. Imipenem-AgNPs conjugate displayed potent antibacterial effect against *P. aeruginosa* [70], and Naqvi et al. [71] and Masri et al. [72] proved the synergistic effect when they also studied different types of antibiotics after their conjugation to AgNPs against several pathogenic microorganisms. Furthermore, kanamycin antibiotic presented capping ability to AuNPs providing a potent antimicrobial agent against some Gram-negative and Gram-positive bacteria [73]. Ciprofloxacin-conjugated ZnO NPs have demonstrated potent antibacterial effect against clinical isolates of *E. coli*, *S. aureus* and *Klebsiella* sp. [68]. The higher synergistic effect mostly noted in the current study could be related to the speculation that NPs enhance the activity of ampicillin and cell penetration since the antibiotic alone showed no activity. NPs might facilitate the ampicillin penetration or binding ability with the cell wall since its bactericidal action is via suppression of cell wall synthesis. AgNPs can anchor to the bacterial cell wall resulting in structural changes such as a membrane permeability increment [74] and therefore facilitate the antibiotic entrance leading to cell death due to their combined activities. However, a significant reduction in the antibacterial effect of the antibiotic-L-NPs conjugate in relation to NPs activity was noted. Further screening and tests are required to study several microbes.

## 4. Conclusions

This investigation has demonstrated for the first time that aqueous extracts from *Leucophyllum frutescens* and *Russelia equisetiformis* are excellent reductants and capping agents in NPs fabrication. Structural analysis of the efficiently produced NPs was confirmed by DSL, TEM and EDS approaches. MTT assays exhibited the NPs’ potential ability in cancer cell suppression as well as their mechanism in cell apoptosis was also noted via TEM and LSM analysis. Ultrastructural changes such as deformed cell shapes, leakage of cell components, large and shrunk nuclei and dead cells were noted. Furthermore, the biogenic NPs showed potential antibacterial ability some against clinical isolates of Gram positive and Gram-negative bacteria. Amp-NPs conjugate had better ability than the NPs alone, suggesting better synergistic effect. Synthesis of NPs using medicinal plants extracts could be beneficial to produce therapeutic agents for the treatment of human diseases. However, more studies are needed to screen the NPs’ efficiency on large scale tumor cells to identify their exact therapeutic effect and furthermore, to validate the synergetic effect of the antibiotic-nanocomposite in order to treat MDR organisms.

## Figures and Tables

**Table 1:** Biogenic nanoparticle size and potential and IC_50_ values (µg/mL) against different cell lines.

Treatment		DLS Measurements		Cancer Cell		Normal Cell
	PDI	Size (d.nm)	Zeta Potential (mV)	MDA-MB-231	HCT116	MCF10A
L-AgNPs	0.252	112.9	−35.1	8.898	22.77	55.48
R-AgNPs	0.214	151.7	−33.7	3.341	12.70	9.731

**Table 2:** Minimum Inhibitory Concentration (MIC) and Minimum Bactericidal Concentration (MBC) of L-AgNPs and R-AgNPs against the tested bacteria.

	L-AgNPs			R-AgNPs		
Microbes	MIC (mg/mL)	MBC (mg/mL)	MIC/MBC	MIC (mg/mL)	MBC (mg/mL)	MIC/MBC
*S. aureus*	0.5	0.8	0.62	0.5	0.8	0.62
*S. mutans*	0.5	0.8	0.62	0.5	0.8	0.62
*E. coli*	0.8	1.1	0.72	0.8	1.1	0.72
*K. pneumoniae*	0.8	1.1	0.72	0.8	1.1	0.72

## Data Availability

Data of this manuscript are displayed in Figure 1, Figure 2, Figure 3, Figure 4, Figure 5, Figure 6, Figure 7, Figure 8, Figure 9, Figure 10, Figure 11, Figure 12, Figure 13, Figure 14, Figure 15, Figure 16 and Figure 17. The facts and raw data analysed are available from the corresponding author upon request.
